# Clinical-CT mismatch defined NIHSS ≥ 8 and CT-ASPECTS ≥ 9 as a reliable marker of candidacy for intravenous thrombolytic therapy in acute ischemic stroke

**DOI:** 10.1371/journal.pone.0251077

**Published:** 2021-04-30

**Authors:** Hung-Ming Wu, I-Hui Lee, Chao-Bao Luo, Chih-Ping Chung, Yung-Yang Lin

**Affiliations:** 1 Institute of Brain Science, National Yang-Ming University, Taipei, Taiwan, Republic of China; 2 Department of Neurology, Taipei Hospital, Ministry of Health and Welfare, New Taipei City, Taiwan, Republic of China; 3 Department of Neurology, Neurological Institute, Taipei Veterans General Hospital, Taipei, Taiwan, Republic of China; 4 School of Medicine, National Yang-Ming University, Taipei, Taiwan, Republic of China; 5 Department of Radiology, Taipei Veterans General Hospital, Taipei, Taiwan, Republic of China; 6 Brain Research Center, National Yang-Ming University, Taipei, Taiwan, Republic of China; 7 Department of Critical Care Medicine, Taipei Veterans General Hospital, Taipei, Taiwan, Republic of China; 8 Institute of Physiology, National Yang-Ming University, Taipei, Taiwan, Republic of China; 9 Institute of Clinical Medicine, National Yang-Ming University, Taipei, Taiwan, Republic of China; Hospital Dr. Rafael A. Calderón Guardia, CCSS, COSTA RICA

## Abstract

**Background:**

Clinical-diffusion mismatch between stroke severity and diffusion-weighted imaging lesion volume seems to identify stroke patients with penumbra. However, urgent magnetic resonance imaging is sometimes inaccessible or contraindicated. Thus, we hypothesized that using brain computed tomography (CT) to determine a baseline “clinical-CT mismatch” may also predict the responses to thrombolytic therapy.

**Methods:**

Brain CT lesions were measured using the Alberta Stroke Program Early CT Score (ASPECTS). A total of 104 patients were included: 79 patients with a baseline National Institutes of Health Stroke Scale (NIHSS) score ≥ 8 and a CT-ASPECTS ≥ 9 who were defined as clinical-CT mismatch-positive (P group) and 25 patients with an NIHSS score ≥ 8 and a CT-ASPECTS < 9 who were defined as clinical-CT mismatch-negative (the N group). We compared their clinical outcomes, including early neurological improvement (ENI), early neurological deterioration (END), delta NIHSS score (admission NIHSS—baseline NIHSS score), symptomatic intracranial hemorrhage (sICH), mortality, and favorable outcome at 3 months.

**Results:**

Patients in the P group had a greater proportion of favorable outcome at 3 months (p = 0.032) and more frequent ENI (p = 0.038) and a greater delta NIHSS score (p = 0.001), as well as a lower proportion of END (p = 0.004) than those in the N group patients. There were no significant differences in the incidence rates of sICH and mortality between the two groups.

**Conclusions:**

Clinical-CT mismatch may be able to predict which patients would benefit from intravenous thrombolysis.

## Introduction

The term penumbra refers to a region of hypoperfused but potentially salvageable brain tissue in cases of acute ischemic stroke [1]. Identifying the penumbra is very important for selecting patients who will benefit the most from intravenous (IV) recombinant tissue plasminogen activator (rtPA) therapy, which is the most effective treatment for acute ischemic stroke within 3 hours of symptom onset [2]. A mismatch between a larger perfusion-weighted imaging lesion (PWI) and smaller diffusion-weighted imaging (DWI) lesion may be used to identify ischemic penumbra [3]. However, the application of PWI prior to IV-rtPA therapy is limited because the use of PWI is time-consuming and often not feasible [4]. Meanwhile, the National Institutes of Health Stroke Scale (NIHSS), which is used to quickly assess stroke severity, is more highly correlated with PWI volume than DWI volume [5]. A large enough mismatch between the NIHSS score and DWI volume is called a clinical-diffusion mismatch (CDM, defined as an NIHSS score ≥ 8 and a DWI volume ≤ 25 mL) [4]. Patients with CDM have a higher probability of infarction growth and early neurological deterioration without thrombolytic therapy [4].

The Alberta Stroke Program Early CT Score (ASPECTS) semi-quantitatively assesses early ischemic changes on computed tomography (CT) and divides the middle cerebral artery (MCA) territory into 10 divisions [6]. One point is subtracted for each division that demonstrates ischemic changes, with a score of 10 indicating no early ischemic changes and a lower score indicating larger ischemic changes in the territory of the MCA [6]. The ASPECTS scores of PWI-DWI mismatch provided high sensitivity and specificity in predicting the volume of PWI-DWI mismatch [7]. The use of the DWI-ASPECTS score instead of the DWI volume successfully predicts that CDM-positive patients (an NIHSS score ≥ 8 and a DWI-ASPECTS score ≥ 8) have a higher risk of early neurologic deterioration than CDM-negative patients (an NIHSS score ≥ 8 and a DWI-ASPECTS score < 8) [8]. After thrombolytic therapy, CDM-positive patients have a greater proportion of favorable outcomes than CDM-negative patients [9]. The CDM could predict the outcomes in patients with acute ischemic stroke [10] and seems to be useful for identifying patients with ischemic penumbra.

Although DWI is commonly used in the assessment of acute stroke, some patients receiving rtPA are contraindicated for urgent magnetic resonance imaging (MRI) [11,12]. Brain CT is the most available method for evaluating patients with hyperacute stroke prior to thrombolytic therapy. Therefore, the CT-ASPECTS score could be more valuable than the DWI-ASPECTS score for identifying patients with ischemic penumbra. A previous study that used a different hypothesis of clinical-CT mismatch (ASPECTS-residual threshold, the difference between each patient’s “expected” ASPECTS calculated by each NIHSS score and the actual ASPECTS at baseline) did not successfully identify the patients who would benefit the most from IV-rtPA therapy [13]. Patients with an NIHSS score < 8 usually have a DWI lesion volume < 25 mL [4], and those patients have no significant difference between their mean PWI volume and mean DWI volume [14]. In contrast, among patients with an NIHSS score ≥ 8, the mean PWI volume was significantly larger than the corresponding DWI volume [14]. A DWI lesion volume of < 25 mL was previously found to correspond to a DWI-ASPECTS score ≥ 8 [8]. Furthermore, the difference in mean baseline ASPECTS score between CT and DWI was found to be approximately 1 to 2 [11]. Therefore, the present study aimed to use a higher CT-ASPECTS value (≥ 9) to evaluate the outcomes of patients who received IV-rtPA therapy. We hypothesized that, in the patients who received IV-rtPA therapy, the patients defined as clinical-CT mismatch-positive (that is, those with an NIHSS score ≥ 8 and a CT-ASPECTS score ≥ 9) had better outcomes than the patients defined as clinical-CT mismatch-negative (that is, those with an NIHSS score ≥ 8 and a CT-ASPECTS score < 9).

## Materials & methods

### Study population

This study was a part of our previous one [15], which retrospectively enrolled acute ischemic stroke patients with symptoms in the anterior circulation who received IV-rtPA therapy at the Neurological Institute of Taipei Veterans General Hospital between January 2012 and November 2017. Clinical data were obtained retrospectively from our database in May 2018. The inclusion and exclusion criteria were the same as those used in our previous study [15]. All patients underwent a brain CT scan before IV-rtPA therapy and 24 hours after IV-rtPA therapy. The ASPECTS score was used to assess the grading of early ischemic changes on pretreatment CT [6]. Stroke severity was assessed using the NIHSS score. One hundred ninety-eight patients with acute ischemic stroke who received IV thrombolysis during the study period met the inclusion criteria. Of those patients, one patient with a pre-stroke mRS score ≥ 1 and 25 patients who also received intra-arterial thrombectomy therapy were excluded. Ultimately, 104 patients with an NIHSS score ≥ 8 were included in the study. Patients with an NIHSS score ≥ 8 and a CT-ASPECTS score ≥ 9 were defined as clinical-CT mismatch-positive, and those with an NIHSS score ≥ 8 and a CT-ASPECTS score < 9 were defined as clinical-CT mismatch-negative. We then divided these patients into P and N groups accordingly.

To explore whether the results might be due to differences between the patients with low CT-ASPECTS scores and those with high CT-ASPECT scores rather than the differences between the clinical-CT-mismatch-positive and clinical-CT-mismatch-negative groups, we also compared the patients by dichotomizing CT-ASPECTS scores into a score of ≥ 7 versus. < 7 or ≥ 8 versus < 8 or ≥ 9 versus < 9.

The use of clinical-CT mismatch is most beneficial for patients with clinically severe stroke in our hypothesis. Therefore, we selected patients with severe (NIHSS score >15) stroke symptoms in the subgroup analysis. In this subgroup, patients with an NIHSS score >15 and a CT-ASPECTS score ≥ 9 were defined as clinical-CT mismatch-positive, and those with an NIHSS score >15 and a CT-ASPECTS score < 9 were defined as clinical-CT mismatch-negative.

The study was approved and the requirement to obtain a signed consent form for all patients was waived by the Institutional Review Board of Taipei Veterans General Hospital (2018-06-014AC). All the methods were performed in accordance with the ethical standards laid down in the Declaration of Helsinki.

### Image acquisition and interpretation

CT scans were performed using a spiral multidetector CT scanner (Brilliance 40, Philips Medical Systems, Cleveland, OH, USA). Individual scans were acquired using contiguous axial 6-mm sections. CT scans were performed using the inferior orbitomeatal line. The scanner settings were 120 kV, 400 mA, and 200 mA. The section time was set to 2 s. The photographs were taken at a window width and level settings of 80/40 HU. The CT-ASPECTS value of each scan was determined by two neurologists/neuroradiologists who were blinded to the background data and clinical outcomes of the patients. If there was any discrepancy in the CT-ASPECTS score, the CT-ASPECTS score was re-evaluated or discussed by two other neurologists/neuroradiologists to reach an agreement.

### Clinical assessment and outcome measurement

We compared the two groups in terms of the following variables: age, gender, history of atrial fibrillation (AF), hypertension (HT), diabetes mellitus (DM), congestive heart failure/left ventricular (LV) dysfunction, previous stroke, or transient ischemic attack (TIA), vascular disease (i.e., prior myocardial infarction, peripheral artery disease, or aortic plaque), smoking, use of anti-platelet or anti-coagulation therapy before stroke onset, glucose level, vital signs and laboratory data including systolic and diastolic blood pressure, international normalized ratio (INR) of prothrombin time (PT), glucose level at entry, NIHSS score prior to thrombolytic therapy and after discharge, the time interval between stroke onset and treatment, the time interval between stroke onset and brain CT scan, and rtPA dose (i.e., the ratio of dose/body weight).

The outcomes were evaluated in terms of symptomatic intracranial hemorrhage (sICH, any type of intracranial hemorrhage with an NIHSS score increased by more than 4 points) during admission, all-cause mortality, early neurological improvement (ENI, a reduction in NIHSS score by ≥ 10 points from baseline or an absolute score ≤ 4 during admission) [16], early neurological deterioration (END, an increase in the NIHSS score by ≥ 1 point from the baseline NIHSS score during admission) [17], delta NIHSS score (the difference in NIHSS score between admission and baseline, admission NIHSS score—baseline NIHSS score), favorable functional outcome defined as an mRS score ≤1 at discharge and 1 month and 3 months after stroke onset, and the Barthel index (BI) at discharge and 1 month and 3 months after stroke onset.

### Statistical analysis

We used the Windows SPSS package (version 20.0, IBM Corp.) to perform the statistical analyses. The chi-square test was used to test the differences in the categorical variables; that is, gender, AF, HT, DM, congestive heart failure/LV dysfunction, previous stroke or TIA, vascular disease, smoking, anti-platelet or anti-coagulation therapy before stroke onset, mRS score ≤1 at discharge, mRS score ≤1 at 1 month, mRS score ≤1 at 3 months, mortality, sICH, ENI, and END. The Kolmogorov-Smirnov test was used to evaluate the data distribution of continuous variables. We used an independent t-test for variables with parametric distributions, such as age, glucose, systolic blood pressure, diastolic blood pressure, and the time interval between stroke onset and treatment. The Mann-Whitney U test was used for variables with non-parametric distributions, such as INR of prothrombin time, rtPA dose, baseline NIHSS score, discharge NIHSS score, BI at discharge, BI at 1 month, and BI at 3 months. All numerical data are presented as mean ± standard deviation (SD). We performed multivariate linear regression analysis to explore the effects of the presence of clinical-CT mismatch on the delta NIHSS score (dependent variables) after adjusting for age, gender, baseline NIHSS scores, CT-ASPECTS scores, and AF. Multivariate logistic regression was used to estimate the independent predictors of favorable outcome. Associations were calculated using the odds ratio (OR) with two-sided 95% confidence intervals (CIs). The kappa value was used to assess the inter-rater and intra-rater reliabilities of the CT-ASPECTS value interpretations. Statistical significance was set at p < 0.05.

## Results

### Baseline characteristics

**Fig 1:**
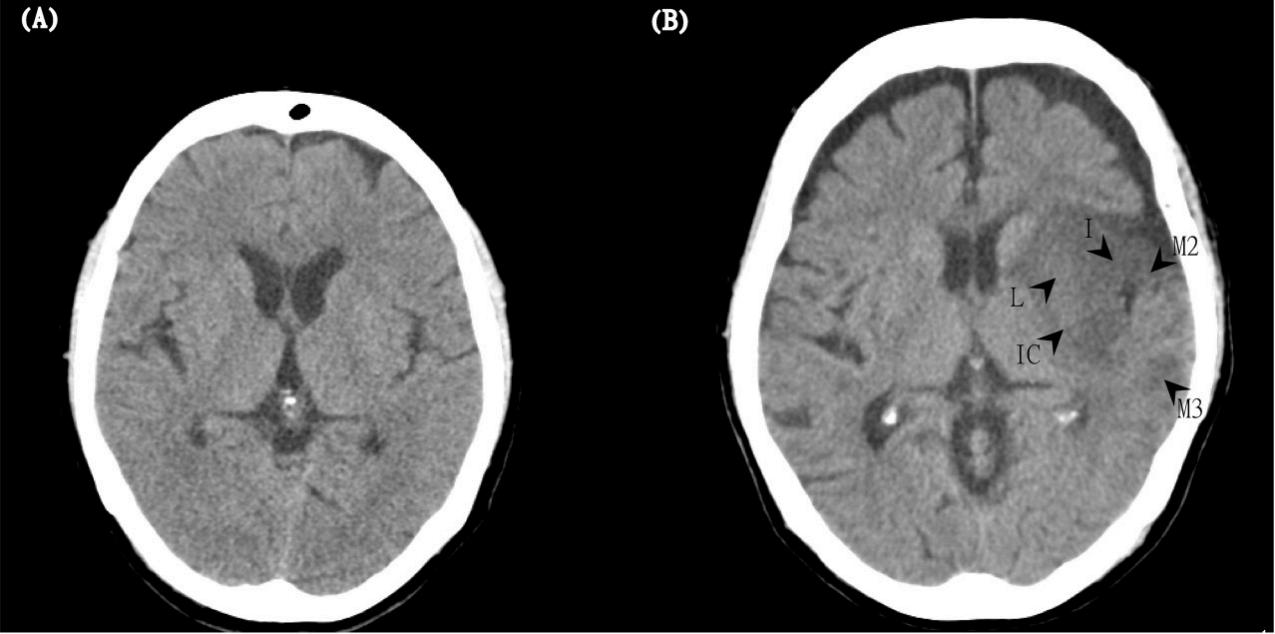
Two representative images of the clinical-CT-mismatch-positive (P group) and clinical-CT-mismatch-negative (N group). (A) The left panel (the P group) shows a 67-year-old woman with an NIHSS score of 16 and a CT-ASPECTS score of 10 at baseline. After IV thrombolysis, the patient had a good outcome (mRS = 0) at 3 months. (B) The right panel (N group) shows an 81-year-old woman with an initial NIHSS score of 22 and a CT-ASPECTS score of 5. Arrows show hypoattenuation in regions of the lentiform nucleus (L), internal capsule (IC), insula (I), M2, and M3. Although she received IV thrombolytic therapy, her mRS score was 5 at 3 months.

There were 79 and 25 patients in the P and N groups, respectively. Representative images of the clinical-CT-mismatch-positive and clinical-CT-mismatch-negative are shown in Fig 1. There were no differences between the two groups in background characteristics, vital signs, and laboratory data, the time interval between stroke onset and treatment, or rtPA dose. However, the baseline and discharge NIHSS scores of the patients in the P group were significantly lower than those of the patients in the N group (P group: 14.34 ± 5.57; N group: 19.52 ± 4.44; p < 0.001) (Table 1). The CT-ASPECTS value analysis of the present study yielded intra-rater and inter-rater reliabilities of 0.743 and 0.608, respectively.

**Table 1:** Clinical characteristics of patients with cerebral infarction receiving intravenous thrombolysis as classified by clinical-CT mismatch.

	Clinical-CT mismatch-positive (n = 79)	Clinical-CT mismatch-negative (n = 25)	p
Age (years), mean ± SD	72.01 ± 13.42	68.24 ± 15.21	0.239
Female, n (%)	30 (38%)	14 (56%)	0.163
Medical history, n (%)			
Atrial fibrillation	24 (30.4%)	12 (48.0%)	0.147
Heart failure	9 (11.5%)	2 (8.0%)	1
Hypertension	53 (67.1%)	18 (72.0%)	0.806
Diabetes mellitus	25 (31.6%)	9 (36.0%)	0.807
Hyperlipidemia	36 (45.6%)	7 (28.0%)	0.163
Prior stroke/TIA	14 (17.7%)	4 (16.0%)	1
Prior MI/peripheral artery disease	13 (16.5%)	7 (28.0%)	0.246
Smoking	25 (31.6%)	8 (32.0%)	1
Oral anticoagulants	5 (6.3%)	2 (8.0%)	0.673
Antiplatelets	23 (29.1%)	4 (16.0%)	0.295
Glucose (mg/dL), mean ± SD	149.59 ± 57.52	157.43 ± 70.34	0.589
Blood pressure (mmHg), mean ± SD			
Systolic blood pressure	164.99 ± 30.88	155.44 ± 32.88	0.188
Diastolic blood pressure	87.35 ± 20.14	92.72 ± 30.14	0.309
INR of PT, mean ± SD	1.05 ± 0.10	1.05 ± 0.07	0.568
Onset to CT (mins), mean ± SD	72.43 ± 44.11	76.06 ± 43.90	0.559
Onset to treatment (mins), mean ± SD	132.33 ± 59.85	138.06 ± 42.43	0.704
rtPA dose (mg/kg), mean ± SD	0.69 ± 0.12	0.71 ± 0.11	0.808
NIHSS score, median ± SD			
Baseline	14.34 ± 5.57	19.52 ± 4.44	< 0.001
Discharge	8.79 ± 7.92	15.84 ± 7.24	< 0.001

Note: CT = computed tomography, INR = international normalized ratio, MI = myocardial infarction, NIHSS = National Institutes of Health Stroke Scale, PT = prothrombin time, rtPA = recombinant tissue plasminogen activator, SD = standard deviation, TIA = transient ischemic attack.

### Outcome and multivariate analysis

The patients in the P group had a greater proportion of favorable functional outcome at 3 months (P group: 30.4%; N group: 8.0%; p = 0.032) and higher scores on the Barthel index at discharge (P group: 49.93 ± 36.33; N group: 21.88 ± 25.70; p = 0.002) and 1 month (P group: 52.17 ± 40.34; N group: 22.61 ± 33.26; p = 0.007) and 3 months (P group: 61.16 ± 38.14; N group: 37.73 ± 36.90; p = 0.014) than the patients in the N group. ENI was more frequent among the patients in the P group than in the N group (P group: 22.8%; N group: 4.0%; p = 0.038), and END was more frequent among the patients in the N group than in the P group (P group, 13.9%; N group, 44.0%; p = 0.004). Delta NIHSS score (P group: -3.78 ± 5.49; N group: -0.76 ± 2.73; p = 0.001) was greater in the patients in the P group than the patients in the N group. However, there were no significant differences in the incidence rates of sICH and mortality between the two groups (Table 2).

**Table 2:** Clinical outcomes of patients with cerebral infarction receiving intravenous thrombolysis as classified by clinical-CT mismatch.

	Clinical-CT mismatch-positive (n = 79)	Clinical-CT mismatch-negative (n = 25)	OR (95% CI)	P
Favorable outcome (mRS ≤1), n (%)				
Discharge	5/79 (6.3%)	0/25 (0.0%)	1.068 (1.008–1.131)	0.334
1 month	16/79 (20.3%)	2/25 (8.0%)	1.157 (0.983–1.354)	0.228
3 months	24/79 (30.4%)	2/25 (8.0%)	1.312 (1.097–1.592)	0.032
Barthel index, mean ± SD				
Discharge	49.93 ± 36.33	21.88 ± 25.70		0.002
1 month	52.17 ± 40.34	22.61 ± 33.26		0.007
3 months	61.16 ± 38.14	37.73 ± 36.90		0.014
Mortality, n (%)	5/79 (6.3%)	2/25 (8.0%)	0.777 (0.141–4.276)	0.673
ICH, n (%)	10/79 (12.7%)	10/25 (40.0%)	0.217 (0.077–0.615)	0.007
sICH, n (%)	3/79 (3.8%)	4/25 (16.0%)	0.207 (0.043–0.999)	0.055
ENI, n (%)	18/79 (22.8%)	1/25 (4.0%)	1.243 (1.076–1.436)	0.038
END, n (%)	11/79 (13.9%)	11/25 (44.0%)	0.651 (0.455–0.931)	0.004
delta NIHSS score, mean ± SD	-3.78 ± 5.49	-0.76 ± 2.73		0.001

Note: CI = confidence interval, CT = computed tomography, END = early neurological deterioration, ENI = early neurological improvement, ICH = intracranial hemorrhage, mRS = modified Rankin scale, OR = odds ratio, sICH = symptomatic intracranial hemorrhage.

Moreover, there was no difference in the favorable outcome at 3 months in patients with ASPECTS ≥ 7 compared with those with ASPECTS < 7, or in patients with ASPECTS ≥ 8 compared with those with ASPECTS < 8 (Table 3). In patients with severe stroke (NIHSS score >15), the baseline NIHSS score was not different between the P and N groups (P group: 20.10 ± 3.94; N group: 20.73 ± 3.10; p = 0.535), but the patients in the P group had a greater proportion of ENI (P group: 19.4%; N group: 0.0%; p = 0.035) and a greater delta NIHSS score (P group: -4.19 ± 6.22; N group: -0.55 ± 2.44; p = 0.013) than the patients in the N group (Table 4).

**Table 3:** Favorable outcome (modified Rankin scale ≤1) at 3 months: Dichotomizing ASPECTS scores into clinical-CT Mismatch-positive or clinical-CT Mismatch-negative based on a score of ≥ 7 versus < 7; ≥ 8 versus < 8; and ≥ 9 versus < 9.

	Clinical-CT mismatch-positive	Clinical-CT mismatch-negative	P
	ASPECTS ≥ 7 (n = 94)	ASPECTS < 7 (n = 10)	
Favorable outcome, n (%)	25 (26.6%)	1 (10%)	0.445
	ASPECTS ≥ 8 (n = 89)	ASPECTS < 8 (n = 15)	
Favorable outcome, n (%)	24 (27.0%)	2 (13.3%)	0.346
	ASPECTS ≥ 9 (n = 79)	ASPECTS < 9 (n = 25)	
Favorable outcome, n (%)	24 (30.4%)	2 (8.0%)	0.032

Note: ASPECTS = Alberta Stroke Program Early CT Score, CT = Computed Tomography.

**Table 4:** Clinical outcomes of patients with severe (NIHSS > 15) stroke symptoms receiving intravenous thrombolysis: classification by clinical-CT mismatch.

	Clinical-CT mismatch-positive (n = 31) in patients with NIHSS > 15	Clinical-CT mismatch-negative (n = 22) in patients with NIHSS > 15	P
mRS ≤1, n (%)	6 (19.4%)	1 (4.5%)	0.218
Mortality, n (%)	2 (2.5%)	2 (9.1%)	1
ICH, n (%)	9 (29.0%)	10 (45.5%)	0.256
sICH, n (%)	2 (6.5%)	4 (18.2%)	0.219
ENI, n (%)	6 (19.4%)	0 (0%)	0.035
END, n (%)	6 (19.4%)	10 (45.5%)	0.068
delta NIHSS score, mean ± SD	-4.19 ± 6.22	-0.55 ± 2.44	0.013

Note: CT = Computed Tomography, END = Early neurological deterioration, ENI = Early neurological improvement, ICH = Intracranial hemorrhage, mRS = modified Rankin scale, NIHSS = National Institutes of Health Stroke Scale, sICH = Symptomatic intracranial hemorrhage.

After multivariate linear regression analysis, the factor significantly related to the delta NIHSS score was the presence of clinical-CT mismatch (p = 0.010). Factors such as age (p = 0.063), gender (p = 0.404), AF (p = 0.412), baseline NIHSS scores (p = 0.943), and CT-ASPECTS scores (p = 0.766) were not significantly related to the delta NIHSS score. In patients with an NIHSS score >15, the presence of clinical-CT mismatch (p = 0.005) and age (p = 0.030) were related to the delta NIHSS score, while factors such as gender (p = 0.897), AF (p = 0.328), baseline NIHSS scores (p = 0.417), and CT-ASPECTS scores (p = 0.810) were not significantly related to the delta NIHSS score (Table 5). The predictors of favorable outcome at 3 months as determined by multivariate logistic regression analysis are presented in Table 6. Adjusted for gender, AF, presence of clinical-CT mismatch, and ASPECTS scores, age, and baseline NIHSS score were predictors of favorable outcome at 3 months.

**Table 5:** Multivariate linear regression analysis: The effect of factors on delta NIHSS score (admission NIHSS—baseline NIHSS score).

	NIHSS ≥ 8		NIHSS > 15	
	β coefficient	*P*	β coefficient	*P*
clinical-CT mismatch	-0.253	0.010	-0.370	0.005
AF	0.080	0.412	0.132	0.328
Age	0.179	0.063	0.284	0.030
Female	0.081	0.404	0.017	0.897
NIHSS, baseline	-0.007	0.943	0.105	0.417
ASEPCTS	0.051	0.766	-0.054	0.810

Note: AF = atrial fibrillation, ASPECTS = Alberta Stroke Program Early CT Score, CT = Computed Tomography, NIHSS = National Institutes of Health Stroke Scale.

**Table 6:** Univariate and multivariate logistic regression analysis for predicting the favorable outcome (mRS ≤1).

	univariate regression		multivariate regression	
	OR (95% CI)	P	Adjusted OR (95% CI)	P
Clinical-CT mismatch	5.018 (1.095–22.999)	0.038	19.021 (0.639–566.583)	0.089
AF	0.261(0.082–0.831)	0.023	0.672 (0.175–2.575)	0.562
Age	0.935 (0.902–0.970)	<0.001	1.080 (1.034–1.129)	0.001
Female	0.809 (0.326–2.005)	0.647	1.012 (0.335–3.055)	0.984
NIHSS, baseline	0.868 (0.788–0.955)	0.004	1.125 (1.003–1.261)	0.044
ASPECTS	1.327 (0.921–1.911)	0.128	1.356 (0.627–2.936)	0.439

Note: AF = atrial fibrillation, ASPECTS = Alberta Stroke Program Early CT Score, CI = confidence interval, CT = computed tomography, NIHSS = National Institutes of Health Stroke Scale, OR = odds ratio.

## Discussion

In the present study, the patients in the P group had a greater proportion of favorable functional outcomes at 3 months, more frequent ENI, less frequent END, and a greater delta NIHSS score than the patients in the N group. There were no significant differences in the incidence rates of sICH and mortality between the two groups.

Only the clinical-CT mismatch, defined as a CT-ASPECTS score ≥ 9, demonstrated the benefit of IV-rtPA therapy. The CT-ASPECTS score ≥ 9 is approximately equal to the DWI-ASPECTS score ≥ 8 according to the difference between CT-ASPECTS and DWI-ASPECTS [11]. A DWI-ASPECTS score ≥ 8 DWI lesion corresponded to a DWI lesion volume < 25m [8]. The specificity of perfusion-diffusion mismatch is decreased in patients with a DWI lesion volume > 25 mL [14]. Therefore, CT-ASPECTS < 9 could decrease the specificity of the perfusion-diffusion mismatch. In other words, CT-ASPECTS = 8 and CT-ASPECTS = 7 have less specificity for perfusion-diffusion mismatch than CT-ASPECTS = 9 to 10. The failure of a previous study to show the benefits of thrombolytic therapy in patients with clinical-CT mismatch might be related to the wider range of CT-ASPECTS (a CT-ASPECTS score ≥7 and an NIHSS score ≥ 8) [13]. It is possible that most patients who receive IV-rtPA have a CT-ASPECTS score ≥ 7 [18], and the wider CT-ASPECTS range might overestimate the patient with penumbra due to decreased specificity of the perfusion-diffusion mismatch.

In the present study, the favorable outcome at 3 months was associated with baseline NIHSS score and age according to multivariate analysis. After thrombolytic therapy, the NIHSS score is a significant factor associated with recanalization within 24 hours [19] and independent status at 3 months [20]. Although lower baseline NIHSS scores have also been found in patients with CDM-positive than those with CDM-negative in a previous study [9], it is reasonable to suspect that the better outcomes in the P group patients were caused by their lower baseline NIHSS scores rather than the clinical-CT mismatch. Therefore, in the subgroup analysis of patients with severe stroke symptoms, based on a similar baseline NIHSS score between the P and N groups, the patients in the P group still demonstrated better outcomes than those in the N group. The clinical-CT mismatch was also a factor associated with the delta NIHSS score (improvement of stroke symptoms) in patients with severe stroke symptoms. The reason might be that the patients with CDM had a lower artery occlusion rate [9] and that the higher ASPECTS scores could predict a higher recanalization rate after thrombolysis [21]. This finding could indicate that the presence of clinical-CT mismatch is important in patients with severe stroke symptoms, which might reflect the finding that a higher NIHSS score has a higher specificity for prediction of the perfusion-diffusion mismatch by CDM [14].

A lower baseline NIHSS score predicts a lower risk of END [22]. However, although patients with CDM had lower baseline NIHSS scores [8,9], they had a higher risk of END when they did not receive thrombolytic therapy [8,14]. In contrast, after thrombolytic therapy, patients with CDM have been found to experience dramatic neurological improvement at 24 h [9]. Perfusion-diffusion mismatch also predicts a higher risk of END without thrombolytic therapy [23]. Moreover, after thrombolytic therapy, patients with perfusion-diffusion mismatch had a higher recanalization rate [24], and reperfusion improved the chance of neurological improvement [25]. CDM within 3 hours after stroke onset predicted good outcomes of thrombolytic therapy [10], whereas CDM from 3 to 6 h after stroke onset did not [25]. In a previous study, the disagreement between CT-NIHSS mismatch and perfusion-diffusion mismatch [18] could be related to the timing discrepancy between the image investigations. The presence and volume of perfusion-diffusion mismatch [26] and the specificity and positive predictive value of CDM-detected perfusion-diffusion mismatch decrease over time [14]. The earlier brain CT scans (i.e., within 4.5 h after stroke onset) conducted in the present study might have increased the degree of overall change observed to better detect the patients with penumbra. However, the timing-related specificity and positive predictive value of clinical-CT mismatch for predicting perfusion-diffusion mismatch are still unclear and warrant further study in the future.

There are some limitations to the present study. First, the investigated cohort of 104 patients was small. Second, selection bias might have been present because this was a retrospective, single-center study. Third, because the ASPECTS scores only assess the lesions within the contralateral MCA territory, the impact of the lesions outside the contralateral MCA territory (the anterior cerebral artery territory or the ipsilateral MCA territory) on outcomes were not considered. Fourth, there was a lack of information on whether the patients had a large vessel occlusion, which could influence clinical recovery in the first 24 hours.

## Conclusions

In conclusion, the results of the present study suggest that clinical-CT mismatch could provide a reliable means of determining acute ischemic stroke patients with penumbra and allow selection of those patients who would benefit more from IV thrombolytic therapy, especially in patients with severe stroke symptoms. In addition, the clinical-CT mismatch was a factor of the delta NIHSS score (reduction of stroke symptoms between baseline and admission). In the future, clinical-CT mismatch might also be useful in choosing alternative treatment strategies for ischemic stroke patients who are evaluated beyond the time window for IV thrombolytic therapy.

## Supporting information

S1 Table Raw data in this study. (XLSX)
